# Development of WEE2 kinase inhibitors as novel non-hormonal female contraceptives that target meiosis^†^

**DOI:** 10.1093/biolre/ioaa097

**Published:** 2020-06-12

**Authors:** Carol B Hanna, Deepti Mudaliar, Kristen John, C Leigh Allen, Luxin Sun, Jon E Hawkinson, Ernst Schönbrunn, Gunda I Georg, Jeffrey T Jensen

**Affiliations:** 1 Oregon National Primate Research Center, Beaverton, Division of Reproductive & Developmental Sciences OR, USA; 2 University of Minnesota, Department of Obstetrics & Gynecology, Minneapolis, MN, USA; 3 Moffitt Cancer Center, Drug Discovery Department, Tampa, FL, USA; 4 Oregon Health & Science University, Portland, OR, USA

**Keywords:** meiosis, WEE2 kinase, oocyte maturation, fertilization, non-hormonal contraceptive

## Abstract

WEE2 oocyte meiosis inhibiting kinase is a well-conserved oocyte specific kinase with a dual regulatory role during meiosis. Active WEE2 maintains immature, germinal vesicle stage oocytes in prophase I arrest prior to the luteinizing hormone surge and facilitates exit from metaphase II arrest at fertilization. Spontaneous mutations at the *WEE2* gene locus in women have been linked to total fertilization failure indicating that selective inhibitors to this kinase could function as non-hormonal contraceptives. Employing co-crystallization with WEE1 G2 checkpoint kinase inhibitors, we revealed the structural basis of action across WEE kinases and determined type I inhibitors were not selective to WEE2 over WEE1. In response, we performed in silico screening by FTMap/FTSite and Schrodinger SiteMap analysis to identify potential allosteric sites, then used an allosterically biased activity assay to conduct high-throughput screening of a 26 000 compound library containing scaffolds of known allosteric inhibitors. Resulting hits were validated and a selective inhibitor that binds full-length WEE2 was identified, designated GPHR-00336382, along with a fragment-like inhibitor that binds the kinase domain, GPHR-00355672. Additionally, we present an in vitro testing workflow to evaluate biological activity of candidate WEE2 inhibitors including; (1) enzyme-linked immunosorbent assays measuring WEE2 phosphorylation activity of cyclin dependent kinase 1 (CDK1; also known as cell division cycle 2 kinase, CDC2), (2) in vitro fertilization of bovine ova to determine inhibition of metaphase II exit, and (3) cell-proliferation assays to look for off-target effects against WEE1 in somatic (mitotic) cells.

## Teaser

Oocyte specific WEE2 is critical for exit from metaphase II arrest in mammalian oocytes and selective inhibitors to this kinase can be developed into non-hormonal contraceptives for women that prevent fertilization.

## Introduction

Currently, the most effective oral approaches to “on-demand” or emergency contraception are hormone-based, and must be administered prior to the onset (levonorgestrel, LNG) or peak (ulipristal acetate, UPA) of the luteinizing hormone (LH) surge to block ovulation, as they have no effect on fertilization or implantation [1]. Both approaches affect the timing of the next menses; LNG typically results in earlier bleeding while UPA results in a delay. A non-hormonal, orally or vaginally active agent that would prevent fertilization of the oocyte without affecting menstrual cycles (even with repeated use in the same cycle) would be a game-changing strategy for woman-controlled on-demand contraception. Daily use orally or via a long-acting delivery system such as an implant could also provide regular contraception.

**Figure 1:**
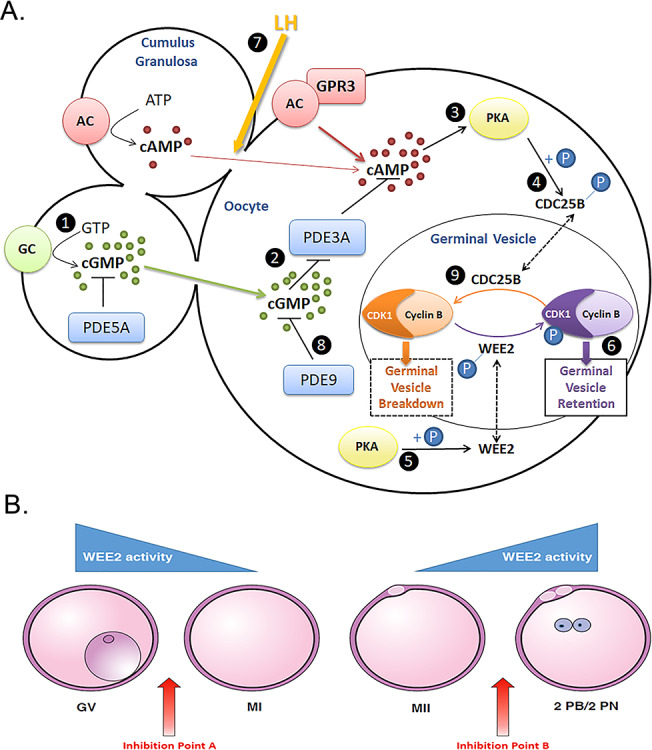
Working hypothesis in developing gamete-based non-hormonal contraceptives that specifically target oocyte meiosis regulation. (A) Cumulus cells surrounding the oocyte synthesize cGMP (1) that diffuses through open gap junctions (GJ) into the oocyte preventing PDE3A (2) from hydrolyzing cAMP. cAMP activates PKA (3), leading to phosphorylation and sequestration of CDC25B to the cytoplasm (4), as well as phosphorylation and translocation of WEE2 kinase to the GV (5). Active WEE2 phosphorylates CDK1, inactivating the CDK1/cyclin B complex (M-phase promoting factor; MPF), and thereby maintaining meiotic arrest (6). Following the LH surge induced gap junction closure (7), PDE9 (8) and PDE3A degrade the cyclic nucleotides necessary for maintaining PKA activity. No longer under suppressive phosphorylation, CDC25B translocates to the GV, where it removes the inhibitory phosphate on CDK1 (9). This leads to MPF activation, GVBD, and resumption of meiosis. (B) Inhibition of WEE2 at point A results in GVBD, resumption of meiosis and no change in fertilization potential. Inhibition at point B results in a failure to exit meiosis, blockade of fertilization, and a contraceptive effect. GV, germinal vesicle; MI, metaphase I; MII, metaphase II; and 2PB/2PN, 2 polar bodies/2 pronuclei.

The oocyte develops fertilization competence during meiosis, the chromosome reduction cell division process that yields gametes. In mammals, primordial germ cells begin meiosis during fetal development, but remain arrested at prophase I in resting (primordial) and growing follicles. The preovulatory surge of gonadotropins induces a cascade of events in the mature follicle leading to resumption of meiosis to yield a fertilizable metaphase II-stage oocyte at the time of ovulation [2]. Oocyte maturation is segregated into nuclear and cytoplasmic events to delineate specific mechanisms and function. Cytoplasmic maturation involves the preparation required for fertilization, activation, and embryo development. Nuclear maturation refers to the meiotic process of chromosomal reduction to a haploid content during resumption of first meiosis following the midcycle LH surge so as to produce a diploid organism upon fusion with sperm. The sentinel sign of reinitiation of meiosis (e.g., nuclear maturation) is germinal vesicle breakdown (GVBD), the dissolution of the nuclear membrane in a process controlled by M-phase promoting factor (MPF), a complex of CDK1 and cyclin B [3, 4]. Cyclic adenosine monophosphate (cAMP) occupies a central role in the regulation of oocyte maturation. Elevated levels of cAMP maintain arrest of the oocyte at prophase I of meiosis by activating protein kinase A (PKA) that in turn phosphorylates oocyte-specific WEE2 permitting translocation into the GV where it in turn phosphorylates CDK1 to suppress MPF activity [5, 6]. The ovulatory LH surge triggers a series of signaling events in the follicle resulting in closure of gap junctions that reverses this inhibition and releases MPF from WEE2 suppression leading to GVBD and the resumption of oocyte maturation to yield an oocyte at the receptive MII state required for fertilization (see Figure 1A).

A number of key enzymes involved in the signaling cascade triggering resumption of meiosis in oocytes have contraceptive potential. Phosphodiesterase (PDE) activity regulates cAMP levels, and inhibitors of PDE3 prevent GVBD in vitro and in vivo and pregnancy in many species including non-human primates [7–9]. While potentially useful as non-hormonal regular contraceptives, the extensive systemic localization of PDE isoforms including PDE3 limits the usefulness of the approach. Also, these pathways must be blocked prior to the LH surge, and offer no advantage for on-demand contraception over existing hormonal approaches.

Notably and in contrast, receptivity to fertilization requires the oocyte to exit from the metaphase II arrest of meiosis, a process again regulated by MPF [10]. Active WEE2 phosphorylates CDK1 decreasing MPF activity and ends meiosis allowing the male and female pronuclei to form, followed by creation of the zygote and initiation of embryonic cleavage events (mitosis). Without WEE2-triggered deactivation of MPF, the oocyte does not respond to sperm penetration, no pronucleus forms, and fertilization fails [5] (Figure 1B). Two WEE kinase members have been identified in mammals: WEE1 and WEE2 (also known as WEE1B). While WEE1 has received considerable attention due to its essential and well-established role in mitosis [11], WEE2 is oocyte specific [12–14]. Thus, selective inhibitors of WEE2 should have contraceptive activity only, and not interfere with embryo development (in the event of failure or improper use) or with function or somatic cells.

## WEE2 in human reproduction

Genetic studies have identified *WEE2* mutations in oocytes as a cause of total fertilization failure (TFF) in women. In 2018, Sang et al. [15] reported the first homozygous mutations at the *WEE2* gene locus leading to infertility in four women seeking clinical reproductive assistance. All individuals presented with normal menstrual cycles and no evidence of male factor infertility. Oocytes obtained from these women following stimulated cycles appeared morphologically normal, could complete meiosis I, and extrude the first polar body (49/53), but failed to exit meiosis and undergo normal fertilization following intracytoplasmic sperm injection (ICSI). The investigators performed whole-exome capture on peripheral blood samples and identified a missense mutation in one women and insertion or deletion (indel) homozygous mutations in three women all leading to loss-of-function from truncated WEE2 protein. All four mutations occurred on different coding exons that varied in localization to the protein kinase catalytic domain (see Table 1). Interestingly, the investigators achieved phenotypic rescue in oocytes injected with native *WEE2* cRNA prior to ICSI, as indicated by the extrusion of the second polar body and exit from metaphase II arrest, hallmarks of fertilization [16]. Additionally, blastocyst formation occurred in 2/4 (50%) embryos demonstrating a successful transition into embryogenesis (mitosis).

**Table 1:** Summary of *WEE2* mutations reported in women with clinical infertility^a^

Genomic position on chromosome 7	cDNA change	Protein change	Mutation type	Exon	Protein kinase Domain	Reference
141 418 986	c.700G>C	p.Asp234His	Missense	4	Yes	[15]
141 427 183	c.1473dupA	p.Thr493Asnfs*39	Frameshift Insertion	10	No	[15]
141 408 777	c.220_223delAAAG	p.Glu75Valfs*6	Frameshift Deletion	1	No	[15]
141 423 059	c.1006_1007insTA	p.His337Tyrfs*24	Frameshift Insertion	6	Yes	[15]
141 424 832	c.1228C>T	p.Arg410Try	Missense	9	Yes	[21]
141 424 038	c.1184G>A	p.Gly395Glu	Missense	8	Yes	[21]
141 419 011	c.725G>C	p.Arg242Pro	Missense	4	Yes	[21]
141 423 050	c.997T>C	p.Ser333Pro	Missense	6	Yes	
141 424 075	c.1221G>A	p.Asp408Valfs*1	Splicing	8	Yes	[21]
141 408 777	c.220_223delAAAG	p.Glu75Valfs*6	Frameshift	1	No	
141 423 059	c.1006_1007insTA	p.His337Tyrfs*24	Frameshift	6	Yes	[21]
141 424 889	c.1286_1288delGAG	p.Gly429del	Deletion	9	Yes	
141 408 777	c.220_223delAAAG	p.Glu75Valfs*6	Frameshift	1	No	[21]
141 418 884	c.598C>T	p.Arg200X	Nonsense	4	No	
141 418 905	c.619C>T	p.Arg207Cys	Missense	4	Yes	[20]
Not reported	c.293_294ins^a^	p.Pro98ProfsX2	Frameshift Insertion	1	No	[18]
Not reported	c.1576T>G	p.Tyr526Asp	Missense	11	No	[18]
Not reported	c.991C>A	p.His331Asn	Missense	6	Yes	[18]
	c.1304_1307delCCAA	p.Thr435Met fs × 31	Frameshift	9	Yes	
Not reported	c.341_342delAA	p.Lys114Asn fs × 20	Frameshift	1	No	[18]
	c.864G>C	p.Gln288His	Missense	5	Yes	
Not reported	c.1A>G	p.0?	Nonsense	1	No	[18]
	c.1261G>A	p.Gly421Arg	Missense	9	Yes	
Not reported	c.585C>G	p.Lys195Asn	Missense	3	No	[17]
Not reported	c.1228C>T	p.Arg410Trp	Missense	9	Yes	[17]
Not reported	c.1006_1007dup	p.His337Tyrfs*24	Frameshift	6	Yes	[17]
	c.1136-2A>G	p.Gly379Glufs*6/p	Splicing	IVS7	Yes	
		Asp380Leufs*39				
Not reported	c.1006_1007dup	p.His337Tyrfs*24	Frameshift	6	Yes	[17]
Not reported	c.598C>T	p.Arg200Ter	Nonsense	4	No	[19]
	c1319C>G	p.Trp440Ser	Missense	9	Yes	

^a^Individual mutations grouped in similarly colored boxes are from consanguineous families.

Since this initial publication, five other reports have described additional cases of female infertility linked to homozygous and heterozygous mutations in *WEE2* (summarized in Table 1) [17–21]. Interestingly, two separate groups describe a mutation at approximately 1006_1007 resulting in a frameshift at p.His337 in four unrelated individuals, suggesting a high incidence of mutation at this site [17, 21]. Additionally, Dai et al. identified a missense mutation at p.Arg410Trp that lead to a “leaky” blockade of metaphase II exit. In this case, out of 30 MII oocytes collected and fertilized by ICSI, six formed pronuclei (three 2PN, and three 3PN) zygotes but none of the embryos developed beyond a six-cell stage possibly due to impaired fertilization.

In all of these clinical case reports, the morphology of the MII oocytes appears normal. Women also reported regular menstrual cycles and no other medical problems. These suggest that a highly selective WEE2 inhibitor could act as a highly selective and well-tolerated non-hormonal contraceptive agent.

## Material and methods

### ADP-Glo activity assay

Compound inhibition of WEE2 activity was determined using the Promega ADP-Glo Max assay (Cat #V7001; Promega Corporation, Madison, WI). The ADP-Glo assay measures kinase activity by luminescent detection of the ADP formed from cleavage of ATP. For HTS, the Chemdiv Allosteric Kinase Inhibitor Collection (25 812 compounds, 10 μM final) was added to 384-well microplates using an Echo 550 dispenser (Labcyte). Recombinant full-length WEE2 enzyme (Schönbrunn lab, Moffitt Cancer Center, Tampa, FL) in assay buffer (50 mM HEPES, pH 7.5, 10 mM MgCl_2_, 1 mM DTT, 0.01% Triton X-100, 0.1 mg/ml BSA) was added at 150–400 ng/well in 2.5 μL using a multidrop (Thermo) and incubated for 10 min at RT. Poly Ala, Glu, Lys, Tyr (6:2:5:1) peptide substrate (Cat #P60-58; SignalChem, Richmond, BC) was then added at 200 ng/well in 2.5 μL buffer containing 100 μM ATP and incubated for 45 min- at RT. The ADP Glo reagent and kinase detection reagents were then added according to the manufacturer’s instructions and luminescence was measured on an EnSpire multimode plate reader (PerkinElmer, Waltham, MA). Compounds producing greater than 40% inhibition were identified as screening hits. To determine inhibitory potency, cherry-picked and repurchased hit compounds were tested at eight concentrations in duplicate 0.3–100 μM and IC_50_ values were calculated using Prism (GraphPad). MK1775 was used as a positive control in all assays.

### Differential scanning fluorimetry assay

The binding potential of compounds for full-length WEE2 and the WEE2 and WEE1 kinase domains (Schönbrunn lab) was assessed by differential scanning fluorimetry (DSF). Compounds were added in duplicate using the Echo to 384-well PCR plates in dose response at 0.3–100 μM final or a fixed concentration of 100 μM final. Proteins (2 μM final) were added in 10 μL buffer (150 mM NaCl, 1 mM DTT, 50 mM HEPES, pH 7.5) containing SYPRO Orange (5000X; Invitrogen) diluted 1:1000. Protein melt curves were measured using a CFX384 thermal cycler (Bio-Rad Life Science, Hercules, CA) with a temperature ramp of 25–99 °C at 0.2 °C increments, 10 s per increment using the FRET channel (560–580 nm emission). The Δ*T*_m_ values were calculated from the first derivative plots, −d(RFU)/dT vs. temperature, relative to a dimethyl sulfoxide (DMSO) control.

### Enzyme-linked immunosorbent assay

Inhibitor activity against WEE1 and WEE2 were assessed using a CycLex WEE1 Kinase Assay/Inhibitor Screening Kit (Cat# CY-1172; MBL International, Woburn, MA) as previously described [22]. Briefly, either 40 mUnits of human WEE1 protein (Cat# CYE1172; MBL International) or 5 μg of recombinant WEE2 protein (Schönbrunn Lab) were incubated with 1 μM inhibitor and ATP in CDC-coated wells (100 μL total volume) for 60 min at 30 °C in accordance with the manufacturer’s protocol. A primary anti-phospho-tyrosine monoclonal antibody (Cat# PY-39; MBL International) was used to detect only the phosphorylated form of tyrosine 15 of CDK1. Tetramethylbenzidine was used as a chromogenic substrate reacting with the horseradish peroxidase conjugated anti-mouse IgG secondary antibody and colorimetric relative quantification of WEE kinase activity was measured by dual wavelength absorbance at 450/540 nm. Student’s *t*-test was used to determine significant changes in WEE kinase activity from controls with *P* < 0.05.

### Bovine in vitro fertilization

To evaluate effects on WEE2 kinase and exit from metaphase II arrest, bovine oocytes were allowed to resume meiosis and undergo in vitro fertilization (IVF) in the presence of inhibitor as described by Hanna et al. [22]. Specifically, cumulus-enclosed oocytes were co-cultured for 18 h with 0.1 or 1 μM MK-1775 or 1% DMSO (control) in 500 μL BO-IVM medium (IVF Bioscience, Falmouth, UK) at 38.5 °C with 5% CO_2_ in humidified air. Resultant ova were transferred to 500 μL BO-IVF (IVF Bioscience) maintaining corresponding concentrations of inhibitor or DMSO and inseminated with 2 × 10^6^ sperm/mL. Presumptive embryos were transferred to BO-IVC medium (IVF Bioscience) without inhibitor or DMSO 20 h post insemination and evaluated for mitotic cleavage 48 h later. One way ANOVA was performed to determine significant changes in embryonic cleavage between treatments with *P* < 0.05.

### Somatic cell proliferation assay

To determine off-target effects of inhibitors on somatic cell WEE1, a proliferation assay was used as previously reported [22]. Briefly, HEK 293 cells were cultured at 37 °C with 5% CO_2_ in humidified air in DMEM medium (Cat# 30–2003; ATCC, Manassas, VA) with 10% fetal bovine serum (Cat# 30-2020; ATCC) and 0, 0.01, 0.1, or 1 μM of MK-1775. Independent cell cultures were collected at 0-, 48-, 72-, and 96-h post loading with a membrane permeable fluorophore, cell trace violet dye (Cat# C34557; ThermoFisher, Waltham, MA), which diminishes in concentration within the cell after each progressive division. Duplicate samples were collected and fixed for analysis by flow cytometry. Emission of the dye was measured at 405/450 nm in at least 20 000 events.

## Structure–function relationships in the WEE family of kinases

We have previously evaluated the structure–function relationship of human WEE2 along with the other members of the WEE kinase family, WEE1 and MYT1 (PKMYT1) [23]. Purified recombinant full-length proteins and kinase domain constructs differed substantially in phosphorylation states and catalytic competency, suggesting complex activation mechanisms of WEE1 and WEE2 by other kinases. Crystal structures of the kinase domains revealed unique structural features that distinguish WEE1 and WEE2 from MYT1. A conspicuous structural element in both WEE1 and WEE2 is a large loop preceding the Asp-Leu-Gly (DLG) motif of the ATP site. In MYT1 and most other kinases, this loop is reduced to a regular three-residue β-turn. The loops are rich in glutamate and serine residues and predicted to contain a PEST proteolytic cleavage site in WEE1, but not in WEE2. WEE1 contains three additional PEST motifs in the N-terminus while WEE2 contains a single PEST motif, suggesting differential modes of enzyme inactivation and degradation during the cell cycle. Residues comprising the ATP site demonstrate strict conservation between WEE1 and WEE2 except for a D386A substitution in the solvent exposed front specificity pocket. Substantial structural changes occur in the P-loop, which adopts an “open” conformation in WEE1 and a “closed” conformation in WEE2. Previous drug discovery efforts have focused on WEE1 and MK-1775 (also known as AZD1775 or Adavosertib) as the first WEE1 inhibitor in clinical trials for solid tumors [24]. Direct binding studies established similar binding potential of MK-1775 for WEE1 and WEE2, but significantly weaker activity against MYT1. Evaluation of several candidate WEE2 inhibitors of diverse chemical scaffolds using co-crystal structures has revealed the structural basis of inhibitory action across WEE kinases [23]. However, none of these type I inhibitors showed significant selectivity for WEE2 over WEE1.

## Identification of selective WEE2 inhibitors

Prior to the availability of the WEE2 crystal structure, our initial attempts reported by Hanna et al. [22], to identify selective WEE2 inhibitors involved a virtual high-throughput screen of the more than 400 000 compounds available at the Institute for Therapeutics Discovery and Development at the University of Minnesota using a homology model of WEE2 that was constructed from the co-crystal structure of WEE1 with PD352396 (PDB: 3B16). The initial filtered data set identified 225 compounds that docked well in to the WEE2 kinase-binding site that also had good potential for bioavailability. To detect compounds with selectivity for WEE2 over WEE1, we evaluated docking scores into the WEE1 crystal structure, and identified 57 compounds that had a 20% higher docking score for WEE2 than for WEE1. Further evaluation of these compounds led to the identification of nine different scaffolds within this set of 57 compounds. Similarity searches provided seven commercial compounds based on those scaffolds. Using ELISA to evaluate functional inhibitory activity against WEE2 and WEE1, we identified two compounds for further consideration for modification. However, no compounds demonstrated sufficient biologic selectivity for further development [22].

**Figure 2:**
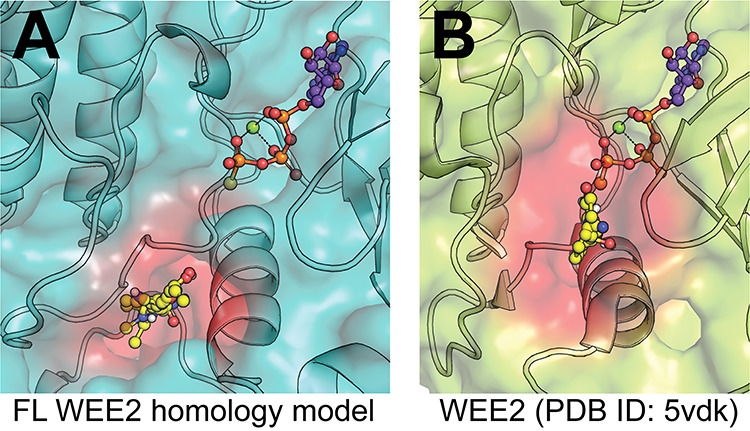
Modeling of potential allosteric site. (A) Full-length WEE2 homology model. FTMap was used on a Phyre2 homology model to identify a region (red) where multiple solvent species (yellow) found energy minima, indicating a potential binding pocket. Orthosteric ATP site is approximated by alignment and extraction of ATPgammaS (non-hydrolyzable ATP analog, purple) from enzyme structure in the same family (EC 2.7.10.2; PDB ID: 5c03). (B) WEE2 kinase domain crystal structure. FTMap was again used to locate potential binding sites in the crystal structure (PDB ID: 5vdk) outside of the orthosteric site, represented by the same ATPgammaS molecule extracted from PDB ID: 5c03 for consistency in orientation. This analysis revealed a similar site (red), which was also identified by FTSite and SiteMap, that engaged with several non-ATP associated side chains.

Due to the similarity of ATP sites in kinases across different families within the kinome, it is often difficult to identify inhibitors with high selectivity for a single kinase. Similar to many other kinases, the reported inhibitors of WEE2 are Type I ATP site inhibitors that lack selectivity for WEE2 over other kinases [23]. One approach to obtain selectivity is to identify allosteric inhibitors, as allosteric binding pockets are restricted to a much smaller subpopulation of kinases. To assess the likelihood of the existence of binding pockets outside the ATP site, a first step involves use of in silico approaches such as FTMap/FTSite and Schrodinger SiteMap to identify potential allosteric sites [25–28]. The FTMap methodology computationally seeks to recapitulate experimental data seen with the labor-intensive multiple solvent crystal structures method to find favorable areas where multiple different solvent probes find low energy groupings. This in silico methodology has been shown to predict allosteric sites in Ras GTPase and other protein targets as well as identify 83% the experimentally confirmed allosteric sites within the three strongest clusters found for a set of G-protein coupled receptors in a recent publication [29, 30]. A full-length homology model of WEE was created using Phyre2 in intensive mode, which incorporated six templates for input into ab initio, multi-template modelling tool Poing, in an effort to predict the N-terminal structure and decrease biasing towards a ligand bound conformation since there are no apo-WEE2 full length structures [31, 32]. Using this model, the FTMap server revealed a hot spot region distal to the active site (Figure 2A), beyond the DLG motif and P-loop, which is proximal to the potential binding site FTMap (Figure 2B), FTSite, and SiteMap identified in the WEE2 crystal structure. Therefore, based on the promising results from FTMap with GPCR allosteric binding predictions, we propose that this area of the protein identified by hot spots in both the ligand-bound crystal structure and the full-length homology model of the apo protein may provide a way to selectively target WEE2 without relying on the orthosteric site. Although few generally applicable methods to identify allosteric inhibitors exist, one approach involves screening for compounds that bind to the target in the hopes of detecting binders outside the ATP site. Techniques such as surface plasmon resonance and DSF can detect binders, however, the former is low throughput and the latter requires relatively large quantities of protein. Although biochemical kinase activity assays preferentially detect ATP site inhibitors, they have the advantage of detecting hits that are inhibitors of the kinase rather than simply binders, which may or may not inhibit the enzyme.

**Figure 3:**
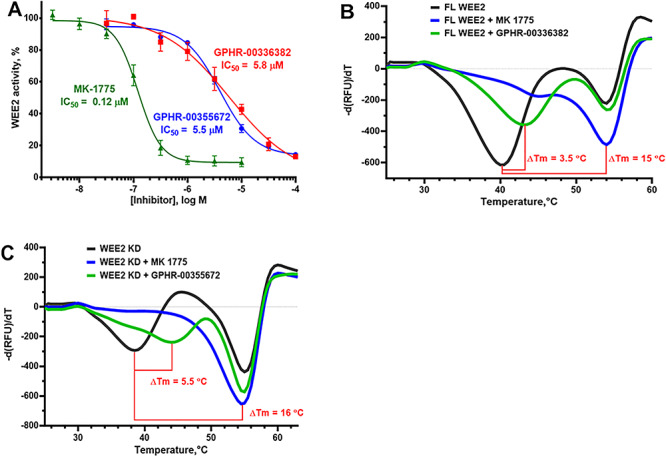
Putative allosteric inhibitors of WEE2. (A) Allosteric kinase inhibitor collection screening hits inhibit WEE2 activity in the ADP-Glo assay with low micromolar IC_50_ values. (B) GPHR-00336382 (100 μM) stabilizes WEE2 with a Δ*T*_m_ of 3.5 °C by DSF. (C) GPHR-00355672 (100 μM) stabilizes WEE2 with a Δ*T*_m_ of 5.5 °C by DSF. MK-1775 was used as a reference ATP site inhibitor in both assays.

We have established an ADP-Glo WEE2 activity assay using a high concentration of ATP (100 μM) to reduce the potency of ATP-competitive inhibitors, thereby biasing the assay towards detecting allosteric inhibitors [33]. Using this allosterically biased activity assay, we conducted a high-throughput screen of a focused library, the ChemDiv Allosteric Kinase Inhibitor collection, containing about 26 000 compounds containing scaffolds known to be allosteric inhibitors of several kinases. Hit confirmation included liquid chromatography-mass spectrometry (LC-MS) and nuclear magnetic resonance (NMR) spectroscopy compound identity and purity greater than 95%, WEE2 ADP-Glo IC_50_ determination [33], lack of interference with the ADP-Glo detection reagents, and lack of redox cycling in the horse radish peroxidase/phenol red assay [34]. We validated hits in orthogonal assays including both ELISA detection of phosphorylated product using a phospho-specific antibody and binding potential by DSF [23]. To confirm allosteric inhibition, hit compound IC_50_ values were determined using three ATP concentrations to confirm the lack of ATP-dependent potency shift as expected for allosteric inhibitors. Using this approach, we have identified a drug-like, selective inhibitor of WEE2 designated GPHR-00336382 (MW 487, WEE2 IC_50_ 5.8 ± 1.2 μM, WEE1 IC_50_ > 100 μM) that binds full-length WEE2 (DSF Δ*T*_m_ 3.5 °C), but not to the kinase domain (Figure 3A and B). We have also discovered a fragment-like selective inhibitor of Wee2 called GPHR-00355672 (MW 287, WEE2 IC_50_ 5.5 ± 0.9 μM, WEE1 IC_50_ > 100 μM) that binds the WEE2 kinase domain (DSF Δ*T*_m_ 5.5 °C) (Figure 3A and C). We have established a preliminary SAR for both hits, and we will attempt to obtain a co-crystal structure for GPHR-00355672 binding to the WEE2 kinase domain.

## Approach to biologic testing of candidate WEE2 inhibitors

Biologic evaluation of candidate WEE2 inhibitors requires assays to confirm (1) disruption of meiotic progression and fertilization in oocytes (e.g., WEE2 inhibition); and (2) absence of an adverse impact on normal mitotic progression in somatic cells (e.g., WEE1/MYT1 inhibition). While cell culture provides a high-throughput approach to screen out agents that disrupt mitosis [35], we lack a high-throughput assay for meiotic inhibition but have developed an in vitro progressive elimination strategy to evaluate candidate WEE2 inhibitors for functional and biological activity.

First, functional inhibitory activity of the compound against WEE1 and WEE2 is determined by sandwich enzyme-linked immunosorbent assay (ELISA). Recombinant WEE proteins are incubated in CDK1 coated plates with ATP and a selected inhibitor to measure the extent of antibody binding to phosphorylated CDK1 (marker of positive WEE activity) and quantified by spectrophotometry measuring a secondary TMB colorimetric reaction. Changes in antibody binding of the CDK1 are compared with controls to survey if the in silico identified compounds display functional inhibition against either WEE1 or WEE2 kinase. Those that are observed to reduce WEE2 activity by at least 80% but have little to no effect on WEE1 activity are moved forward for further assessment.

Following this functional assessment, selected compounds were further evaluated for biological inhibitory activity against WEE2. Bovine oocytes provide a good experimental model for meiosis studies. WEE kinase functions are conserved across species, and human and bovine WEE2 protein share a 73% homology [36]. Additionally, they share similar ovarian cycle length and endocrine activity with women and undergo similar timings of meiosis resumption, fertilization, and embryonic cleavage [37]. To evaluate blockade of fertilization, we collect abattoir derived bovine ovaries and aspirate small antral ovarian follicles, less than 8 mm in diameter, to isolate healthy, presumed GV stage oocytes surrounded by at least two layers of cumulus cells. To control for biologic variability, each collection represents oocytes from multiple animals.

Oocytes are cultured for 18 h with inhibitor to allow for meiosis resumption and evaluated for effects on cumulus expansion before in vitro insemination with bovine sperm and inhibitor to assess for normal fertilization [22]. Embryonic cleavage is observed for day 2 post insemination and any ova which do not cleave can be further validated for fertilization blockade by imaging the nuclear material to determine if pronucleus structures are present (fertilized) or if the ova remains in a metaphase II configuration (not fertilized). While labor intensive, this approach allows for evaluation of multiple treatments and/or concentrations with small groups of oocytes (40–50/group) within each treatment. Promising candidate inhibitors can then undergo further validation in the more translational but expensive non-human primate model [14].

To evaluate the biological off-target effect of candidate inhibitors on mitosis, we use somatic cell culture and flow cytometry to assess proliferation disruption. In these experiments, HEK 293 somatic cells undergo culture with a dose gradient of inhibitors with samples collected at 0, 48, 72, and 96 h. Prior to each culture experiment, we treat the cells with a membrane permeable fluorescent dye to track cellular division activity. As cells divide the dye becomes diluted and the fluorescent signal decreases. We use flow cytometry, with at least 20 000 events counted to measure fluorescence intensity to indicate changes in cell proliferation in the presence of inhibitors [38]. The more inhibitory a drug, the more dye will be retained in the cells and so comparison can be made to controls to determine the level of mitotic activity.

**Figure 4:**
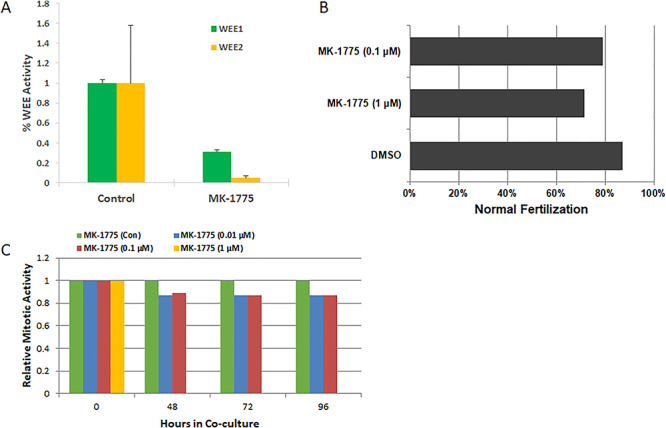
Functional and biological evaluation of WEE inhibitors in oocytes and somatic cells. (A) ELISA analysis for phosphorylation activity of WEE1 and WEE2 in situ. MK-1775 significantly inhibits WEE1 and WEE2, reducing a greater proportion of WEE2 kinase activity. Error bars represent SEM with *P* > 0.05 by Student *t*-test. (B) Fertilization rates of bovine oocytes co-cultured with inhibitor or DMSO (control) during IVF (*N* = 126). MK-1775 reduced MII exit compared to DMSO by 16%. (C) Somatic cell division activity (mitosis) was tracked over time by flow cytometry analysis. MK-1775 induced a decrease in mitosis at 0.1 and 0.01 μM and arrested the cell culture at 1 μM.

We have evaluated MK-1775 as a model compound to validate our progressive elimination strategy and investigate in vitro effects on WEE2 phosphorylation of CDK1, exit from metaphase II arrest at fertilization in vitro using the bovine models, and inhibition of somatic cell proliferation. MK-1775 significantly reduces WEE1 and WEE2 phosphorylation activity of CDK1 (Figure 4A). Incubation of bovine oocytes with MK-1775 at 1 μM prior to and during IVF resulted in a reduction in the proportion of oocytes undergoing normal fertilization (Figure 4B) confirming pharmacologic inhibition, but this concentration also inhibited mitosis in somatic cell culture after 48 h and higher concentrations showed toxicity (Figure 4C).

Following this approach provides a standardized method of consistently validating inhibitors following in silico screening and selection and has been used to successfully identify three potential WEE2 inhibitors from an initial small compound library of over 400 000 for further development into non-hormonal contraceptives [22]. Identification of allosteric inhibitors offers a promising alternative approach with greater specificity across the kinome.

## Conclusions

In this paper, we present a framework for development of novel non-hormonal female contraceptives based on oocyte-specific pathways critical for meiosis from target identification, virtual high-throughput screening, high-throughput screening of focused compound collections, use of X-ray crystallography for evaluation of structural relationships, and medicinal chemistry. Candidate agents identified through this process proceed to cell-free ELISA to confirm activity and specificity. We measured binding by DSF and WEE1 and WEE2 inhibitory activity by both ATP hydrolysis and inhibition of phosphorylation of CDK1. To date, results of these efforts in our WEE2 program have yielded two molecules of interest for further study; a selective inhibitor that binds full-length WEE2 designated GPHR-00336382, along with a fragment-like inhibitor that binds the kinase domain, GPHR-00355672. Finally, we present a strategy for efficient biologic testing of efficacy and specificity: IVF of bovine ova to determine inhibition of metaphase II exit (WEE2 inhibition); and cell-proliferation assays to evaluate off-target effects against WEE1 in somatic (mitotic) cells. We presented results with MK-1775, a model compound with mixed WEE2/WEE1 inhibitory activity to illustrate this approach.

WEE2 kinase, a key cell-cycle regulating protein found only in germ cells (egg and sperm), has great potential as a target for non-hormonal contraception. Blockade of WEE2 prevents fertilization of the oocyte through a non-hormonal mechanism, extending on-demand contraception to a time point much later than currently possible with hormonal methods. A WEE2 inhibitor could also function as an effective non-hormonal method of regular contraception. Identification of frameshift and indel mutations in the *WEE2* gene in women with infertility provide further validation of WEE2 as a contraceptive target.

While a promising target, attempts to develop specific inhibitors targeting WEE2 suffers from the general problems associated with kinase inhibitors. The high similarity in the ATP-binding domain across the kinome presents major challenges in medicinal chemistry. While the elucidation of the crystal structure for WEE2 suggests opportunities for the development of kinase-binding domain (Type I/II) inhibitors, our initial attempts at screening have not succeeded. The development of allosteric inhibitors offers an alternative approach of great interest, with promising leads under evaluation. Early success in the development of allosteric inhibitors has been reported for a limited number of kinases [39–45]. To date, there are five FDA-approved allosteric inhibitors. Two are the type III allosteric inhibitors of MEK1/2: trametinib and cobimetinib, and three additional are FKBP-12 binders that inhibit mTOR: everolimus, temsirolimus, and sirolimus [46]. Recently, a research group from Novartis reported on the development of a novel potent and selective allosteric inhibitor of BCR-ABL1 that binds to the myristoyl pocket [47]. While not yet screened for biological activity against meiotic and somatic cells, allosteric inhibitors represent an alternate class of compounds to develop and evaluate for WEE2 selectivity.

Testing promising candidate agents for activity and specificity requires use of validated bench assays, moving the most promising compounds forward to in vitro evaluation of the relevant biologic endpoints. Proof-of-concept testing of lead compounds in non-human primate models can provide substantial preliminary data to support submission of an investigational new drug application leading to clinical trials in women.

Higher throughput biologic screens would speed the pace of drug discovery. The use of oocytes is expensive and labor intensive, even when using the bovine or mouse model. A somatic cell culture would provide a less expensive alternative, but somatic cells do not express WEE2. Efforts underway include an attempt to integrate a functional *WEE2* gene into a somatic cell line, and to then knockout *WEE1*. An immortalized cell line dependent on WEE2 activity for completion of mitosis would represent a powerful tool for “higher” throughput screening of WEE2 inhibitors.

Comprehensive genetic studies of infertility phenotypes in women with oocyte maturation defects have yielded other potential targets that deserve further attention and screening for druggability as novel contraceptives. In addition to the studies from China that identified *WEE2* mutations as a genetic basis for infertility, TFF has also been associated with mutations in PATL2 [48]. PALT2 functions as an oocyte specific RNA-binding protein [49–51]. Mutations in TUBB8, an oocyte-specific tubulin required to form the meiotic spindle, have also been identified in a cohort of Chinese patients with oocyte maturation defects [50].

The lack of investment by industry and government in novel methods of female contraception greatly hampers progress. New funders like the Bill & Melinda Gates Foundation have stepped up to fill this void. The process will take time and substantial investment. While genetic studies will help guide identification of key pathways essential for fertility with drugable targets, finding suitable candidate drugs still requires considerable expertise and patience. Finding better approaches to high-throughput screening could potentially reduce costs and shorten time lines. Final validation of targets will require testing in animals. Nonhuman primate contraception studies provide an option to test promising approaches and new drugs for safety and efficacy prior to moving these forward to clinical studies in women. Nonhormonal methods that do not block ovulation or thicken cervical mucus, such as WEE2 inhibitors, will require the development of other surrogates for pregnancy risk for early phase studies. While research studies continue to strive for solutions to these hurdles, it is clear selective WEE2 inhibitors represent a unique opportunity to advance the field of non-hormonal contraception and warrants continued support and investment.
